# Inhibiting G protein βγ signaling blocks prostate cancer progression and enhances the efficacy of paclitaxel

**DOI:** 10.18632/oncotarget.16428

**Published:** 2017-03-21

**Authors:** Prakash Paudyal, Qing Xie, Prasanna Kuma Vaddi, Michael D. Henry, Songhai Chen

**Affiliations:** ^1^ The Department of Pharmacology, Roy J. and Lucille A. Carver College of Medicine, University of Iowa, Iowa City, IA 52242, USA; ^2^ The Department of Molecular Physiology and Biophysics, Roy J. and Lucille A. Carver College of Medicine, University of Iowa, Iowa City, IA 52242, USA; ^3^ The Department of Pathology, Roy J. and Lucille A. Carver College of Medicine, University of Iowa, Iowa City, IA 52242, USA; ^4^ The Department of Urology, Roy J. and Lucille A. Carver College of Medicine, University of Iowa, Iowa City, IA 52242, USA; ^5^ The Holden Comprehensive Cancer Center, Roy J. and Lucille A. Carver College of Medicine, University of Iowa, Iowa City, IA 52242, USA; ^6^ The Department of Internal Medicine, Roy J. and Lucille A. Carver College of Medicine, University of Iowa, Iowa City, IA 52242, USA

**Keywords:** G protein-coupled receptors, Gβγ, prostate cancer growth and metastasis, cancer stem cells

## Abstract

Aberrant activation of G protein-coupled receptors (GPCRs) is implicated in prostate cancer progression, but targeting them has been challenging because multiple GPCRs are involved in cancer progression. In this study, we tested the effect of blocking signaling via a hub through which multiple GPCRs converge — the G-protein Gβγ subunits. Inhibiting Gβγ signaling in several castration-resistant prostate cancer cell lines (i.e. PC3, DU145 and 22Rv1), impaired cell growth and migration *in vitro*, and halted tumor growth and metastasis in nude mice. The blockade of Gβγ signaling also diminished prostate cancer stem cell-like activities, by reducing tumorsphere formation *in vitro* and tumor formation in a limiting dilution assay in nude mice. Furthermore, Gβγ blockade enhanced the sensitivity of prostate cancer cells to paclitaxel treatment, both *in vitro* and *in vivo*. Together, our results identify a novel function of Gβγ in regulating prostate cancer stem-cell-like activities, and demonstrate that targeting Gβγ signaling is an effective approach in blocking prostate cancer progression and augmenting response to chemotherapy.

## INTRODUCTION

Prostate cancer is the most common cancer affecting men in the Western World and the second leading cause of cancer death among American men [1]. The five-year survival rate for patients with localized prostate cancer is nearly 100%, but it decreases to 28% for patients in advanced stages with metastases [2]. Androgen-deprivation therapy (ADT) is the standard of care for advanced cases; and although initially effective, most patients with metastatic tumors eventually relapse with castration-resistant prostate cancer (CRPC) [3].

Many mechanisms foster CRPCs. For example, androgen-receptor (AR) signaling can persist, despite low levels of circulating androgen; alternatively, any one of many signaling pathways controlled by receptor tyrosine kinases and G protein-coupled receptors (GPCRs) can become deregulated. Such changes can allow prostate cancer growth to become androgen independent [4–8]. Emerging evidence, however, suggests another source of androgen independent CRPCs may be a small subpopulation of cells that retain stem-like properties [9–11]. These stem-like cells can propagate tumors, have a survival advantage, and escape current chemotherapies, suggesting their stem-like qualities allow them to persist, become drug-resistant, proliferate, and metastasize. These cells have been termed cancer stem cells (CSCs).

CSCs are known to overexpress GPCRs (*e.g*., CXCR4), which when stimulated in prostate cancer cells trigger growth, migration, and invasion [12]. Activated GPCRs might exert their tumorigenic effects directly by stimulating heterotrimeric G-protein-dependent signaling, or indirectly by trans-activating androgen and growth-factor receptors [13]. Thus, overexpressed, activated GPCRs could signal to CSCs to become tumorigenic and chemotherapy resistant [12].

GPCRs are the largest family of cell-surface receptors and desirable drug targets for diverse diseases [14]. Of the more than 350 non-sensory GPCRs, many are overexpressed in prostate cancer and implicated in prostate cancer progression [15, 16]; these include receptors for chemokines (*e.g*., CXCR4 and CXCR7), bradykinin, lysophosphatidic acid (LPA), and endothelin 1 [17–24]. GPCR ligands (e.g., LPA, IL8, and SDF1α) are also secreted at high levels by prostate cancer cells [25–28], where several GPCRs are overexpressed simultaneously, likely cooperating to drive tumor progression via redundant pathways. Redundant GPCR signaling may have led to failure of several clinical trials that targeted single GPCRs (such as those targeting the endothelin 1 receptor) [16, 29, 30]. Thus, to harness the therapeutic power of blocking GPCR signaling, it may be more effective to target a shared pathway downstream of many GPCRs.

Downstream of most GPCRs, signals are transmited through heterotrimeric G proteins, which consist of Gα, Gβ, and Gγ subunits [14]. Gα subunits in their inactive state heterodimerize with Gβ and Gγ subunits [14]. GPCRs activate G proteins by inducing GTP/GDP exchange on Gα subunits, leading to the dissociation of Gα from Gβγ subunits. Both GTP-bound Gα and liberated Gβγ subunits transmit signals to downstream effectors and both are implicated in tumor progression, but accumulating evidence indicates that Gβγ plays a particularly important role in tumor growth and metastasis, therefore representing an attractive therapeutic target [31].

Gβγ subunits, rather than Gαi/o subunits, transmit the primary proliferation signals for a large group of GPCRs overexpressed in prostate tumor cells (such as receptors for chemokines, bradykinin and LPA) [13, 32]. Moreover, in prostate cancer cells, liberated Gβγ likely transactivates EGF receptors [33, 34]. Inhibiting Gβγ has been shown to block prostate cancer PC3 cell growth *in vitro* and primary tumor growth in nude mice [35, 36]. Gβγ also serves as a point of convergence for signals from multiple GPCRs in breast cancer cells, where Gβγ promotes tumor growth and metastasis [37, 38], but whether it plays a similar role in increasing prostate cancer metastasis is unknown. Moreover, it has never been investigated whether Gβγ signaling mediates GPCR activity in increasing prostate cancer CSC tumorigenicity and sensitivity to chemotherapy.

In this study, we showed that inhibiting Gβγ signaling in several castration-resistant prostate cancer cell lines not only blocked progression of preexisting primary prostate tumors but also suppressed formation of tumor metastases in bone and soft tissues. Moreover, we provide evidence that, both *in vitro* and *in vivo*, Gβγ signaling may be involved in maintaining the population and tumorigenicity of prostate CSCs. Furthermore, we showed Gβγ blockade sensitized prostate cancer cells to the chemotherapeutic agent, paclitaxel. Our data thus reveal that prostate cancer CSC tumorigenicity is driven by a novel function of Gβγ signaling, and that targeting Gβγ signaling may be a new way to eliminate these cells to block tumor progression.

## RESULTS

### Expression of Gαt selectively blocks GPCR signaling in prostate cancer cells

**Figure 1:**
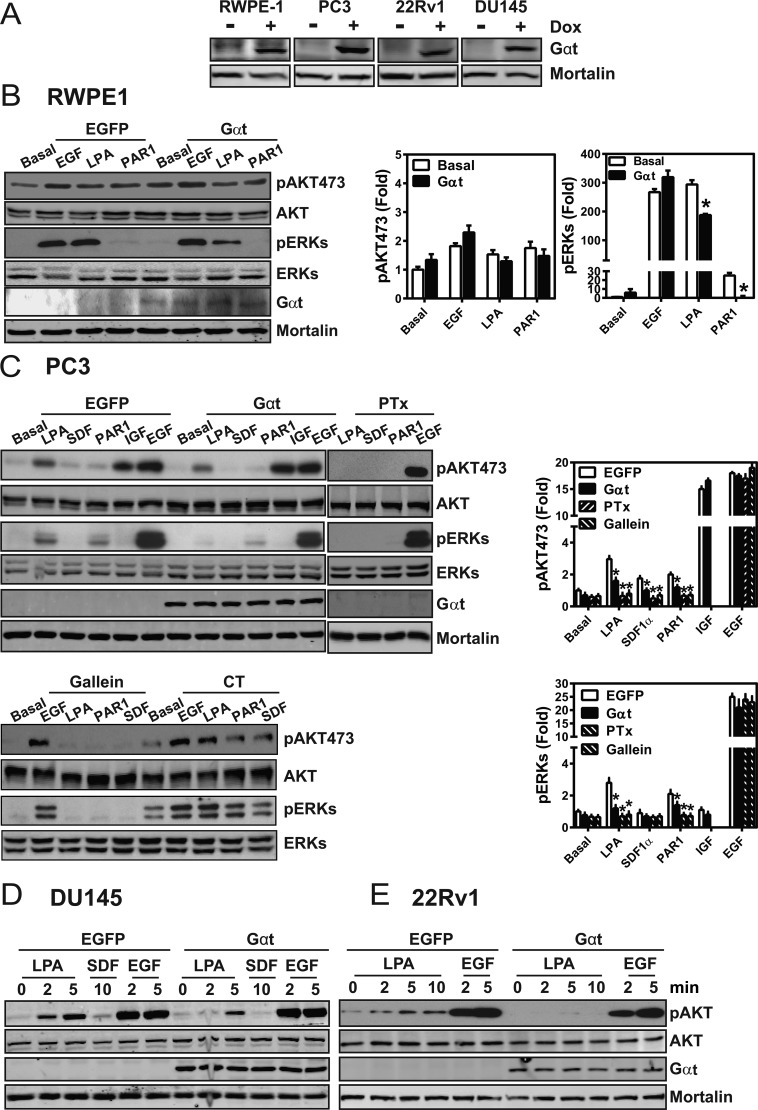
Induced Gαt expression selectively blocks GPCR-mediated signaling in prostate cancer cells (**A**) Gαt expression from doxycycline inducible lentiviruses, in RWPE-1, PC3, 22Rv1 and DU145 cell lines; expression was induced by doxycycline (1 μg/ml) for three days. (**B**–**E**) Gαt expression, pertussis toxin (PTx) or gallein treatment inhibited GPCR signal transduction in RWPE1 (B) prostate cancer cell lines (C–E). Cells were treated with doxycycline for 3 to 5 days to induce transgene expression, overnight with PTx (200 ng/ml), or gallein (20 uM) for 1 hour and then stimulated with LPA (10 μM), SDF1α (50 nM), PAR1 agonist peptide (20 μM), IGF (100 ng/ml) or EGF (20 ng/ml) for 5 min or the indicated times. Representative images and quantitative data from at least three independent experiments are shown.

In our study of prostate cancer cells, Gβγ signaling was manipulated by expressing recombinant Gαt, a specific inhibitor of Gβγ that binds free Gβγ and selectively prevents the activation of Gβγ effectors without interfering with Gα signaling [37, 39]. Here, recombinanat lentiviruses stably transduced castration-resistant prostate cancer cell lines (i.e., PC3, DU145, 22RV1) and an nontransformed prostate epithelial cell line (RWPE1) with tetracycline-inducible expression vectors encoding GFP (control) or Gαt (Figure 1A). To assess how Gαt expression affected Gβγ signaling, we tracked AKT and ERK phosphorylation, in the presence of several GPCR agonists. These GPCR agonists included LPA, SDF1α and a protease-activated receptor 1 (PAR1) peptide agonist, all of which can activate AKT and ERK via Gβγ-dependent mechanisms [37]. As control, cells were stimulated in parallel with IGF or EGF, which activate receptor tyrosine kinases. As shown in Figure 1B–1F, in all cell lines tested, stimulation of GPCRs activated AKT and/or ERK phosphorylation, but the levels of response varied widely in different cell types and also dependent on the types of ligands used. Gαt expression inhibited GPCR-stimulated AKT and ERK phosphorylation in all cancer cell lines tested, but only suppressed GPCR-stimulated ERK but not AKT phosphorylation in RWPE1 cells. IGF and EGF signalling for activated AKT and ERK was unaffected by Gαt expression. Similar results were observed with a selective inhibitor of Gβγ, gallein, in RWPE1 (not shown) and PC3 cells (Figure 1C), suggesting that GPCR stimulated AKT and ERK phosphorylation through a Gβγ-dependent pathway in prostate cancer cell lines, and both Gβγ-independent and dependent pathways in RWPE1 cells. In PC3 cells, LPA-, SDF1α and PAR1-stimulated AKT and ERK phosphorylation was also sensitive to pertussis toxin (Figure 1C). Since pertussis toxin exclusively uncouples Gi/o proteins from their receptors by catalyzing the ADP-ribosylation of the Gai/o subunits [40], these findings suggest that these GPCRs activate AKT and ERK in cancer cell lines primarily through Gβγ freed from Gi/o proteins.

### Gβγ signaling promotes prostate cancer cell growth, proliferation and migration

**Figure 2:**
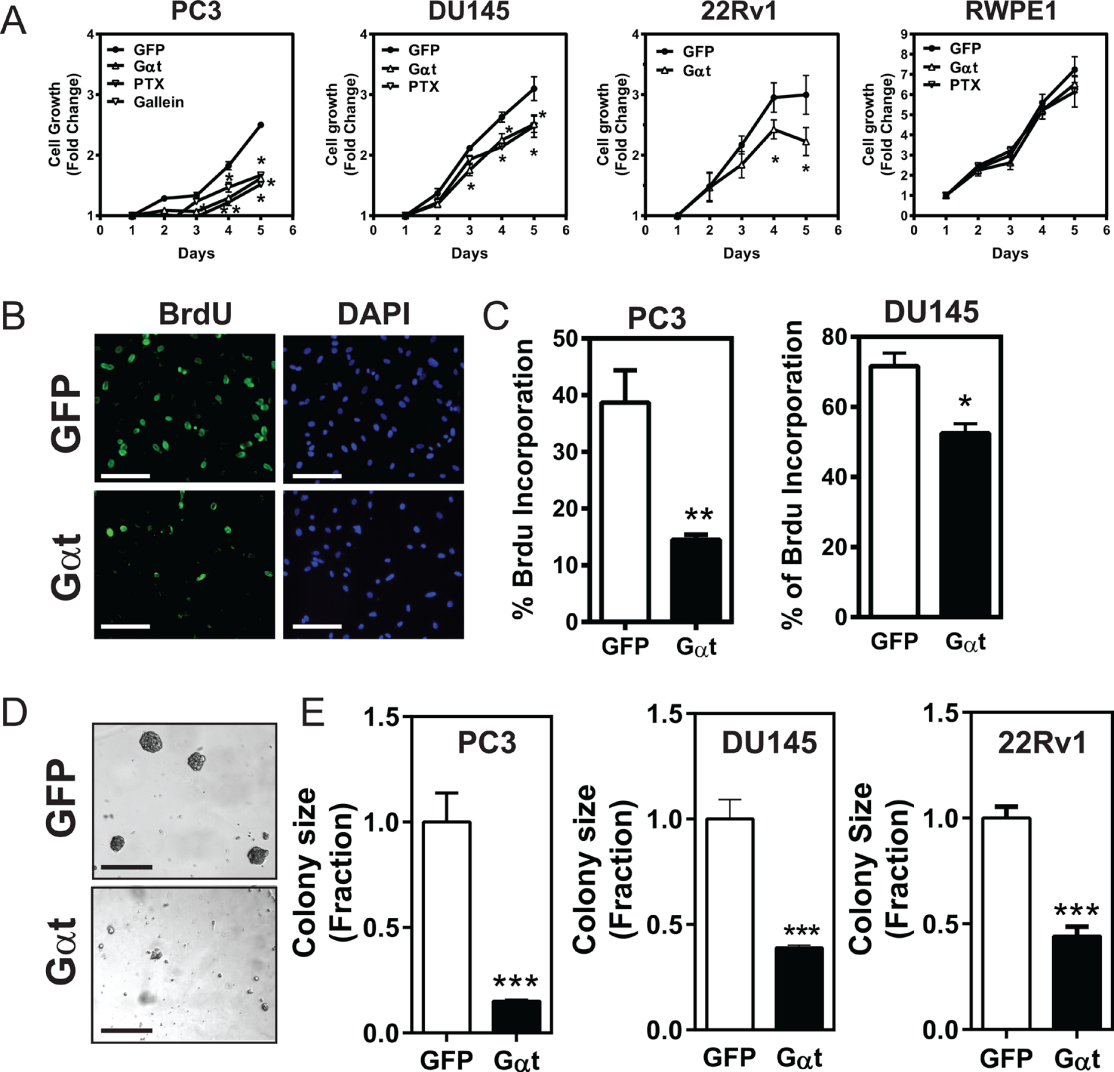
Blocking Gβγ signaling via Gαt decreases prostate cancer cell proliferation The indicated cells were treated with doxycyline (1 μg/ml) to induce transgene (GFP or Gαt) expression. In some cases (**A**), GFP-expressing cells (PC3, DU145 and RWPE1) were also treated with pertussis toxin (PTx; 200 ng/ml) or gallein (PC3; 20 μM). A-C, cell growth in 2D culture was measured by XTT assays (A) or BrdU incorporation assays (**B**, **C**). BrdU incorporation was detected by immunofluorescence staining. Representative fluorescence images of BrdU and nuclear (DAPI) staining in PC3 cells are shown in B. Quantitative data showing BrdU incorporation in PC3 and DU145, expressed as the percentage of cells stained with BrdU (C). *, **p < 0.05 and 0.01 *versus* GFP, respectively (*n* = 3–4). (**D**, **E**) the effect on cell growth in Matrigel was determined by phase-contrast imaging, followed by quantification of the size of the colonies. Colony size is expressed as the fraction of GFP-expressing cells. Representative images of GFP- and Gαt-expressing PC3 cells grown in Matrigel are shown in D. Scale, 100 mm. ***p < 0.001 *versus* GFP (*n* = 3–5).

We then asked how blocking Gβγ signaling affects cell growth. Compared to GFP expression, Gαt expression significantly decreased PC3, DU145 and 22Rv1 cell growth in monolayer culture in a XTT-based cell viability assay and a 5-bromo-2′-deoxyuridine (Brdu) incorporation assay (Figure 2A–2C). The slower proliferation of prostate cancer cells expressing Gαt was also evident by the 50 to 80% reduction in the size of colonies in a 3D-matrigel culture (Figure 2D, 2E). Treatment of prostate cancer cells with PTx reduced proliferation of GFP-expressing cells to a level comparable to that of Gαt-expressing cells (Figure 2A), indicating the primary involvement of Gβγ subunits released from Gi/o proteins. Simuilar to Gαt expression, gallein treatment also reduced PC3 cell growth (Figure 2A). Interestingly, the growth of RWPE1 cells was neither affected by PTx treatment nor Gαt expression (Figure 2A). These findings suggest that Gβγ signaling is critical for the proliferation of prostate cancer cells but is dispensable for the growth of normal, prostate epithelial cells.

**Figure 3:**
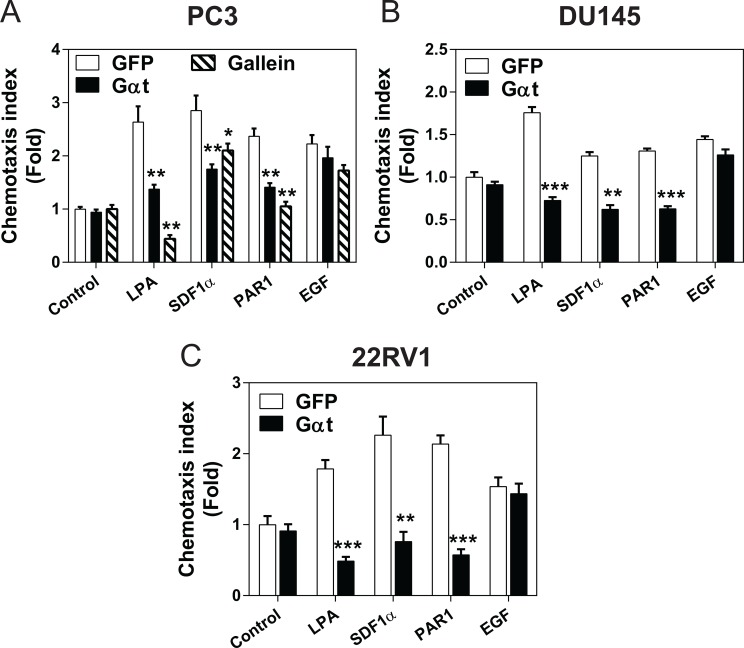
Blocking Gβγ signaling impedes GPCR-induced prostate cancer cell migration GFP or Gαt was induced by doxycycline for 5 days in PC3 (**A**), DU145 (**B**) and 22Rv1 (**C**). In PC3 cells, GPF-expressing cells were also treated with or without gallein (20 μM). The effects on cell migration were determined by a transwell migration assay in response to buffer (control), LPA (10 nM), SDF1α (100 nM), PAR1 agonist peptide (10 μM) or EGF (50 ng/ml). **, ***p < 0.01 and 0.001, respectively, *versus* GFP (*n* = 3–4).

Next, we evaluated the role of Gβγ signaling in prostate cancer cell migration. In a transwell migration assay, the migration of Gαt-expressing PC3, DU145 and 22Rv1 lines toward several GPCR agonists (i.e., LPA, SDF1α, and PAR1) was significantly reduced (Figure 3A–3C). In contrast, these cells migrated normally toward EGF, a response not controlled by Gβγ (Figure 3A–3C). Similarly, GPCR-mediated PC3 cell migration was also inhibited by gallein (Figure 3A).

### Blocked Gβγ signaling impairs prostate tumor growth and metastasis *in vivo*

**Figure 4:**
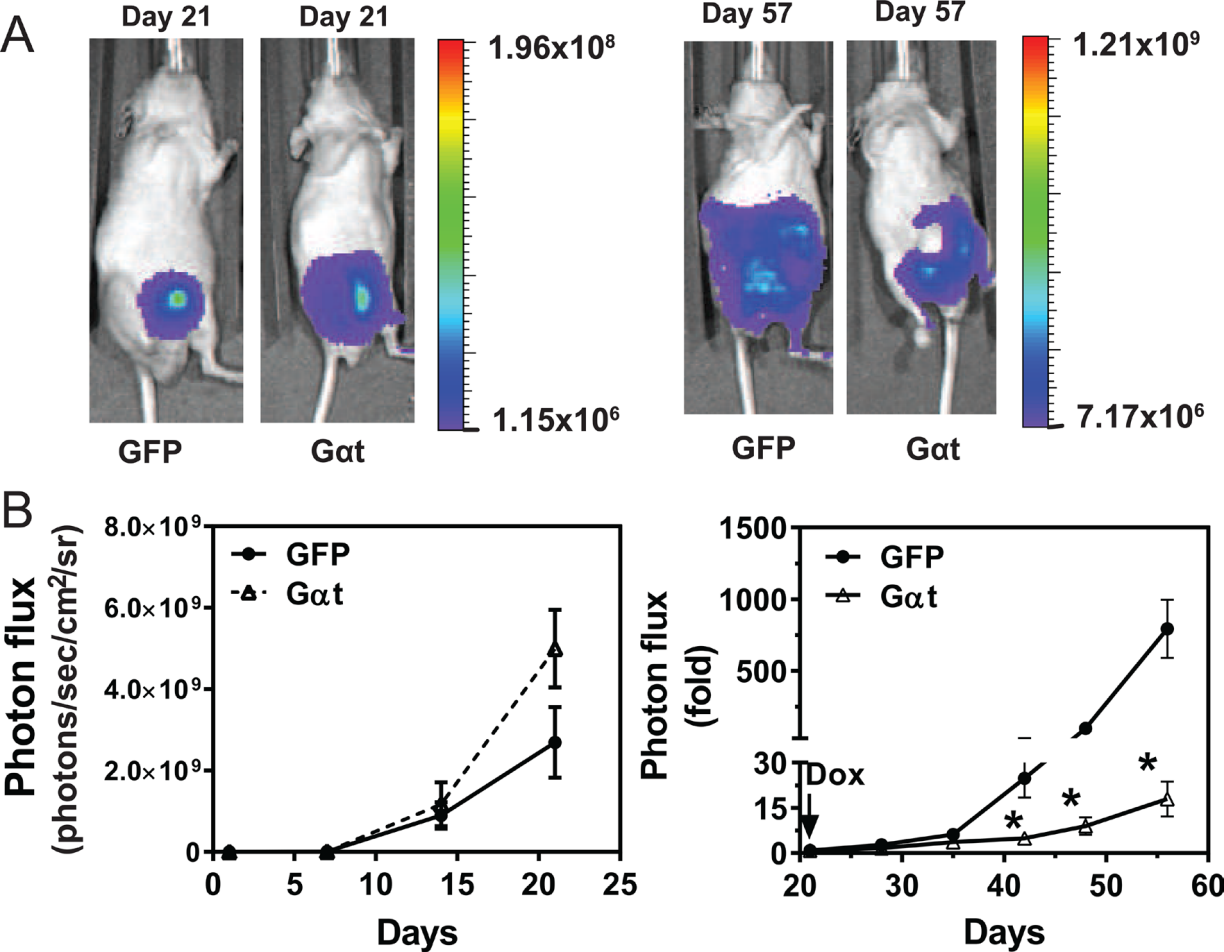
Targeting Gβγ signaling via Gαt blocks primary prostate cancer growth 22Rv1 expressing luciferase and inducible GFP or Gαt were implanted into the prostates of nude mice (*n* = 6). 21 days post implantation, mice were fed doxycycline-containing diets to induce transgene expression. Tumor growth was monitored by bioluminescence imaging. Representative bioluminescence images (**A**) and quantitative data (**B**) of primary tumor growth at the indicated times. After doxycycline-induced GFP and Gαt expression, tumor growth is expressed as fold increase in photon flux over that at day 21.

To examine whether blocking Gβγ signaling inhibits prostate cancer cell growth *in vivo*, 22Rv1 cells expressing inducible GFP or Gαt were injected, in equal numbers, into the prostate gland of nude mice. Without inducing transgene expression, bioluminescence imaging showed Gαt-expressing cells prolifereated at the same rate or slightly faster than GFP-expressing cells (Figure 4A, 4B). After 21d, when transgene expression was induced with doxycycline, significant reduction in tumor growth was seen in mice injected with Gαt-expressing cells, as compared to GFP-expressing cells (Figure 4A, 4B). These findings indicate that Gβγ signaling is critical for maintaining 22Rv1 orthotoptic xenograft growth.

**Figure 5:**
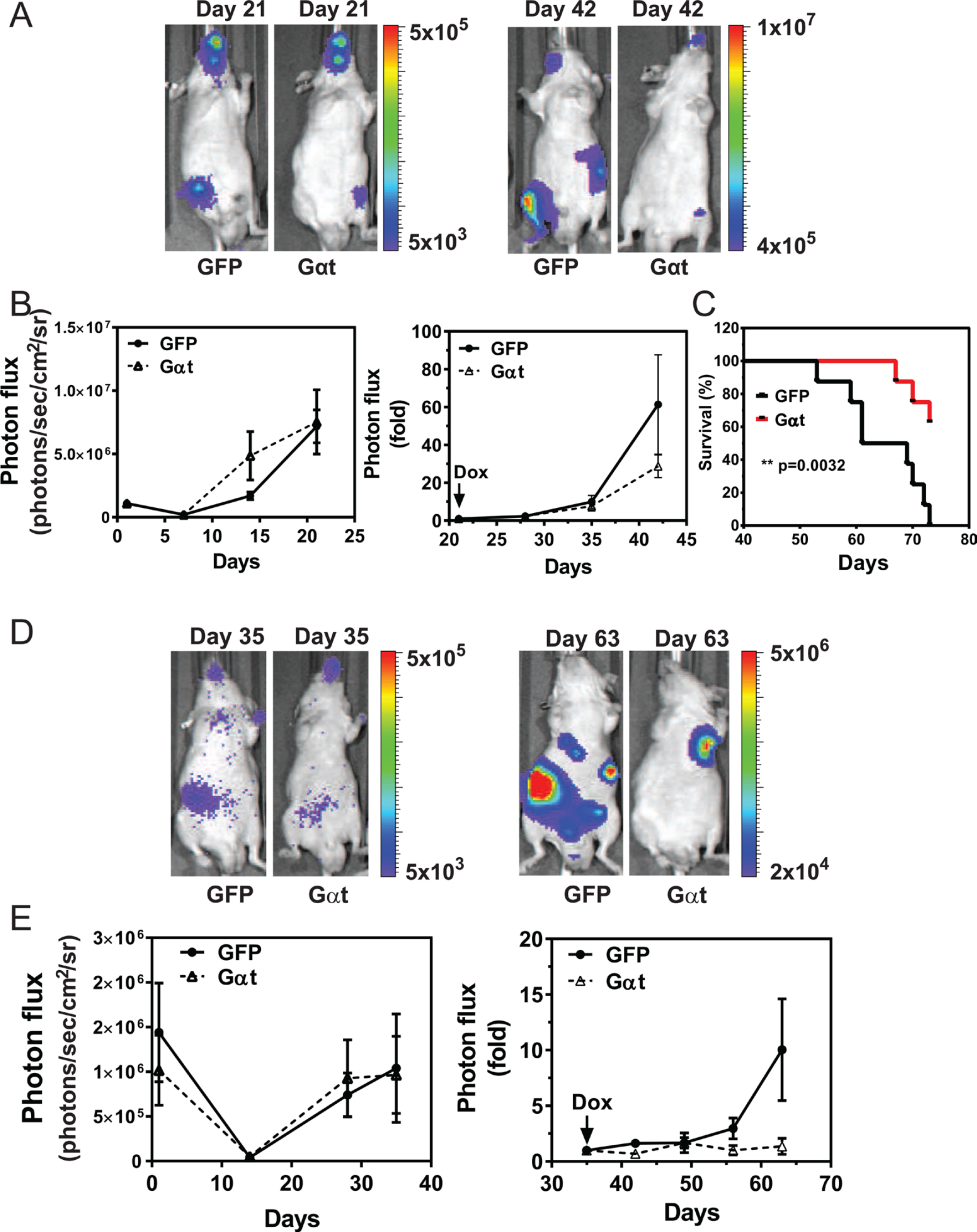
Induced Gαt expression reduces prostate cancer metastasis and increases survival Nude mice (*n* = 6 to 7) were inoculated with 22Rv1 (**A**–**C**) or PC3 (**D**, **E**) cells by intracardiac injection. At 21 (A–C) or 35 (D, E) days post injection, mice were fed doxycycline-containing diets to induce transgene expression. Tumor growth was monitored by bioluminescence imaging. Representative bioluminescence images (A and D) and quantitative data (B and E) of tumor growth at the indicated times are shown. C, overall survival curve of mice inoculated with 22Rv1 cells.

To test if Gβγ signaling drives prostate cancer metastasis, we injected 22Rv1 cells expressing inducible GFP or Gαt into the left ventricle of nude mice, to disseminate tumor cells to multiple organs. Injected cells were allowed to form tumors in the absence of doxycycline induction for 21 days. Over this period, BLI revealed all injected cells grew at comprabe rates, throughout the animals’ bodies (Figure 5A–5C). Upon inducing GFP or Gαt expression, whole-body BLI analysis suggested Gαt-expressing cells proliferated more slowly, but the difference was not statistically significant (Figure 5B). *Ex vivo* BLI, however, revealed that Gαt-expressing cells gave rise to fewer tumors, in multiple organs (i.e., brain, lung, kidney, leg and mandible; Table 1). Moreover, mice bearing Gαt-expressing cells were significantly improved in overall survival (Figure 5C). Similar results were found for PC3 cells (Figure 5D–5E and Table 2). These findings indicate that Gβγ signaling is also critical for the outgrowth of prostate cancer metastases in multiple organs.

**Table 1:** The frequency of 22Rv1 tumor metastasis formation at various tissues of nude mice inoculated with 22Rv1 cells expressing inducible GFP or Gαt via intracardiac injection

Tissues	GFP (*n* = 6)	Gαt (*n* = 6)
Brain	3 (50%)	1 (16.6%)
Lung	1 (16.6%)	0
Kidney	1 (16.6%)	0
Mandible	5 (83.3%)	4 (66.6%)
Femur	1 (16.7%)	1(16.6%)
Tibia	1 (16.7%)	0

The number and percentage of mice detected with tumors at the indicated tissues by *ex vivo* BLI are indicated.

**Table 2:** The frequency of PC3 tumor metastasis formation at various tissues of nude mice inoculated with PC3 cells expressing inducible GFP or Gαt via intracardiac injection

Tissues	GFP (*n* = 7)	Gαt (*n* = 7)
Brain	2 (28.6%)	0 (0%)
Liver	2 (28.6%)	0 (0%)
Lung	3 (42.9%)	1 (14.3%)
Kidney	2 (28.6%)	1 (14.2%)
Spleen	2 (28.6%)	0 (0%)
Mandible	3 (42.9%)	3 (42.9%)
Femur	3 (42.9%)	1 (14.3%)
Tibia	2 (28.6%)	0 (0%)

The number and percentage of mice detected with tumors at the indicated tissues by *ex vivo* BLI are indicated.

### Blocked Gβγ signaling targets aggressive, stem-like cells in prostate tumors

**Figure 6:**
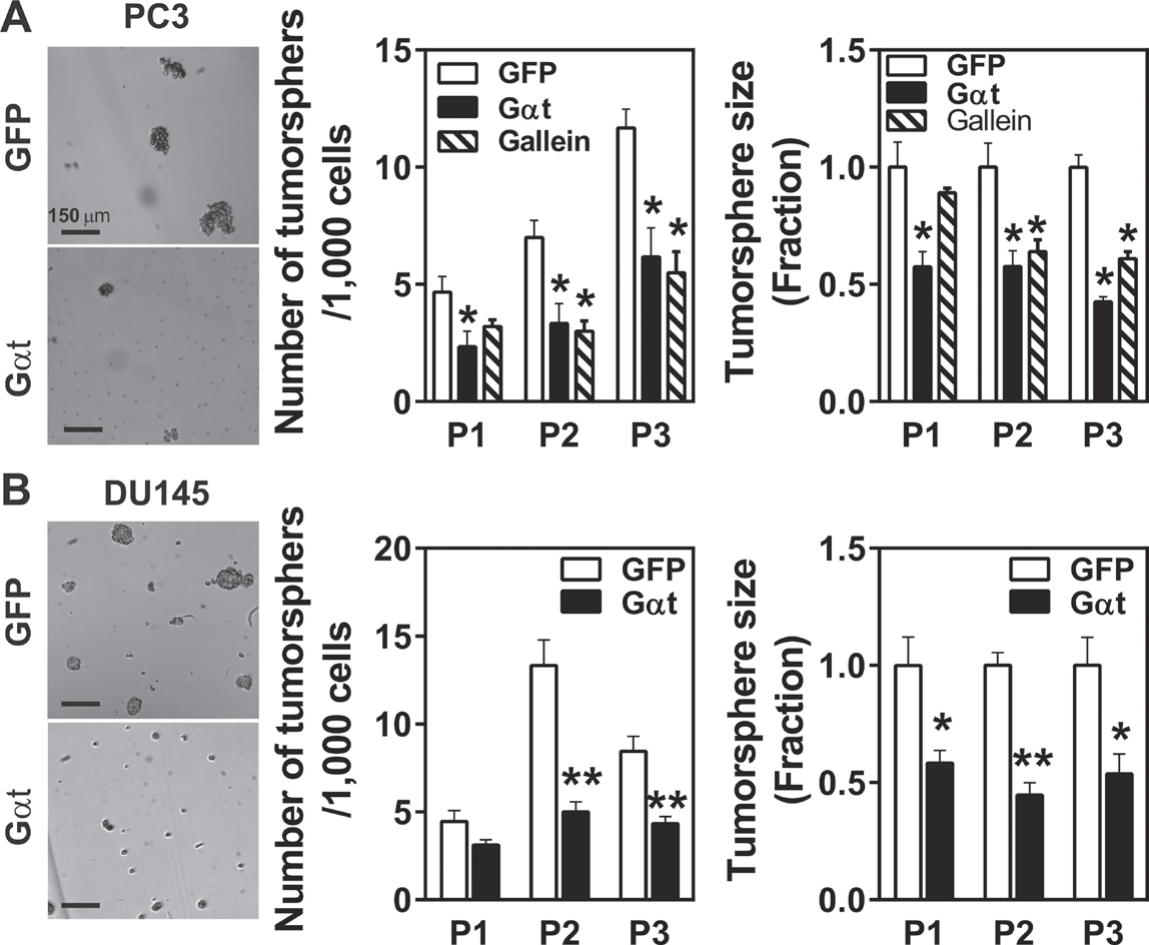
Inhibition of Gβγ signaling decreases tumorsphere-forming capability of prostate cancer cells PC3 (**A**) and DU145 (**B**) cells expressing inducible GFP or Gαt were grown under tumrosphere-forming conditions in the presence of doxycycline (1 ml) or gallein (20 μM) for 8–10 days. Primary tumorspheres from passage 1 (P1) were dissociated by Accutase, and single cells were plated onto ultralow-adhesive plates for further expansion (P2 and P3). The number of tumorspheres was counted under a microscope and the size of tumorsheres was determined by phase-contrast imaging, followed by Image J analysis. *, **p < 0.05 and 0.01 *versus* GFP, respectively, *n* = 3–6.

Prostate cancer cells harbor a small population of CSCs that may contribute to metastasis and recurrence [9]. Given that prostate cancer cell growth and metastasis was robustly inhibited by Gβγ blockade, we tested whether Gβγ signaling regulates the activities of their CSCs. Prostate cancer CSCs can be identified *in vitro* by their ability to grow primary and secondary tumorspheres upon serial passaging under non-attached conditions in ultralow-adhesive plates; and *in vivo* by their ability to generate tumors after serial transplantation into mice [41, 42]. As reported [41, 43], PC3 and DU145 cells generate increasing number of tumorspheres upon serial passaging (Figure 6A, 6B). Inhibiting Gβγ signaling by inducing Gαt expression or gallein treatment decreased the number and size of tumorspheres generated from PC3 and DU145 cells (Figure 6A, 6B). Similar results were found for 22Rv1 cells (data not shown).

To verify these findings *in vivo*, we cultured GFP-expressing and Gαt-expressing PC3 cells, without doxycycline induction, under tumorsphere-forming conditions for three passages to enrich for CSCs. Then GFP-expressing and Gαt-expressing tumorspheres were dispersed into a single-cell suspension and injected subcutaneously, (50,000, 20,000, or 10,000 per mouse) into nude mice. Mice were immediately fed doxycycline-containing diets to induce transgene expression. In parallel, we also treated another group of mice bearing GFP-expressing cells with the Gβγ inhibitor, gallein.

**Figure 7:**
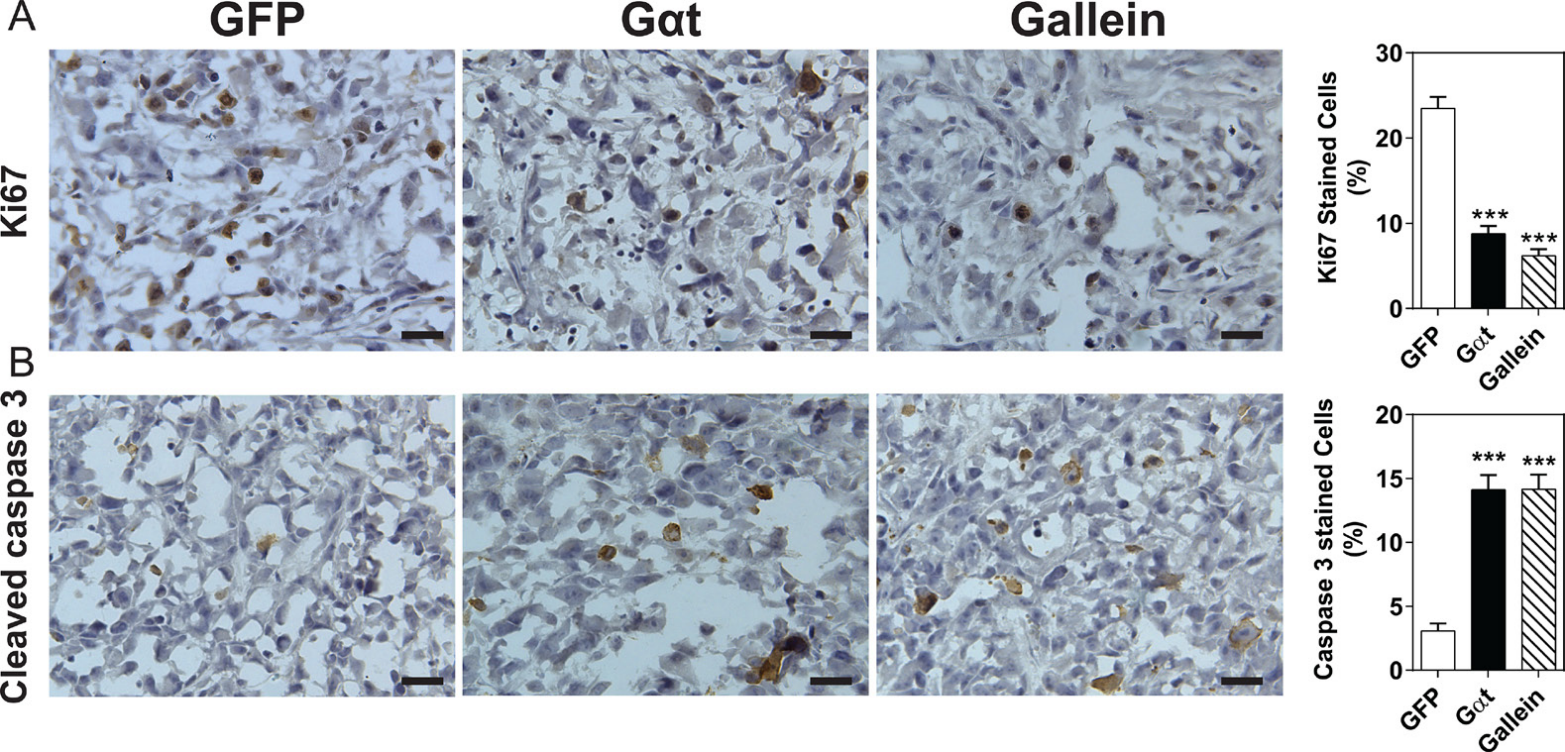
Blocking Gβγ signaling slows proliferation and triggers apoptosis of prostate cancer cells Tumor sections were prepared from tumors derived from mice inoculated with 20,000 PC3 cells, as indicated in Table 3. Sections were stained with Ki67 (A) or cleaved caspase 3 (B). Representative images of the indicated tumor sections and quantitative data are shown. ***p < 0.001 *vs* GFP, *n* = 5.

Two months post injection, all mice that had been inoculated with 50,000 GFP-expressing or Gαt-expressing cells formed palpable tumors (Table 3), but the size of tumors derived from Gαt-expressing cells or gallein-treated mice tended to be smaller (data not shown). Compared to mice inoculated with a comparable number of GFP-expressing cells, mice inoculated with 20,000 and 10,000 of Gαt-expressing cells developed fewer palpable tumors (60 and 80% *vs* 40 and 0%, respectively; Table 3). The frequency of tumor formation in mice inoculated with 10,000 GFP-expressing cells also decreased from 80% to 20% with gallein treatment (Table 3). Immunohistochemical analysis of Ki67 expression and caspase 3 cleavage activation revealed that blocking Gβγ signaling by Gαt expression or gallein treatment significantly reduced prostate cancer cell proliferation and increased apoptosis in the xenograft tumors (Figure 7A, 7B). These findings suggest Gβγ signaling may be required for maintaining and renewing the population of prostate CSCs.

**Table 3:** The frequency of tumor formation in nude mice (*n* = 5) inoculated with the indicated number of single PC3 cells dissociated from the third passages of tumorspheres

Number of cells	50, 000	20, 000	10, 000
GFP	100 %	60 %	80 %
Gαt	100 %	40 %	0 %
GFP+Gallein	100 %	80 %	20 %

Immediately post inoculation of PC3 cells, mice were fed with doxycycline-containing diet to induce GFP or Gαt expression and treated with or without gallein (10 mg/kg, twice daily). Mice were monitored weekly for palpable tumors. Data are expressed as percentage of mice with palpable tumors two month-post innoculation.

**Figure 8:**
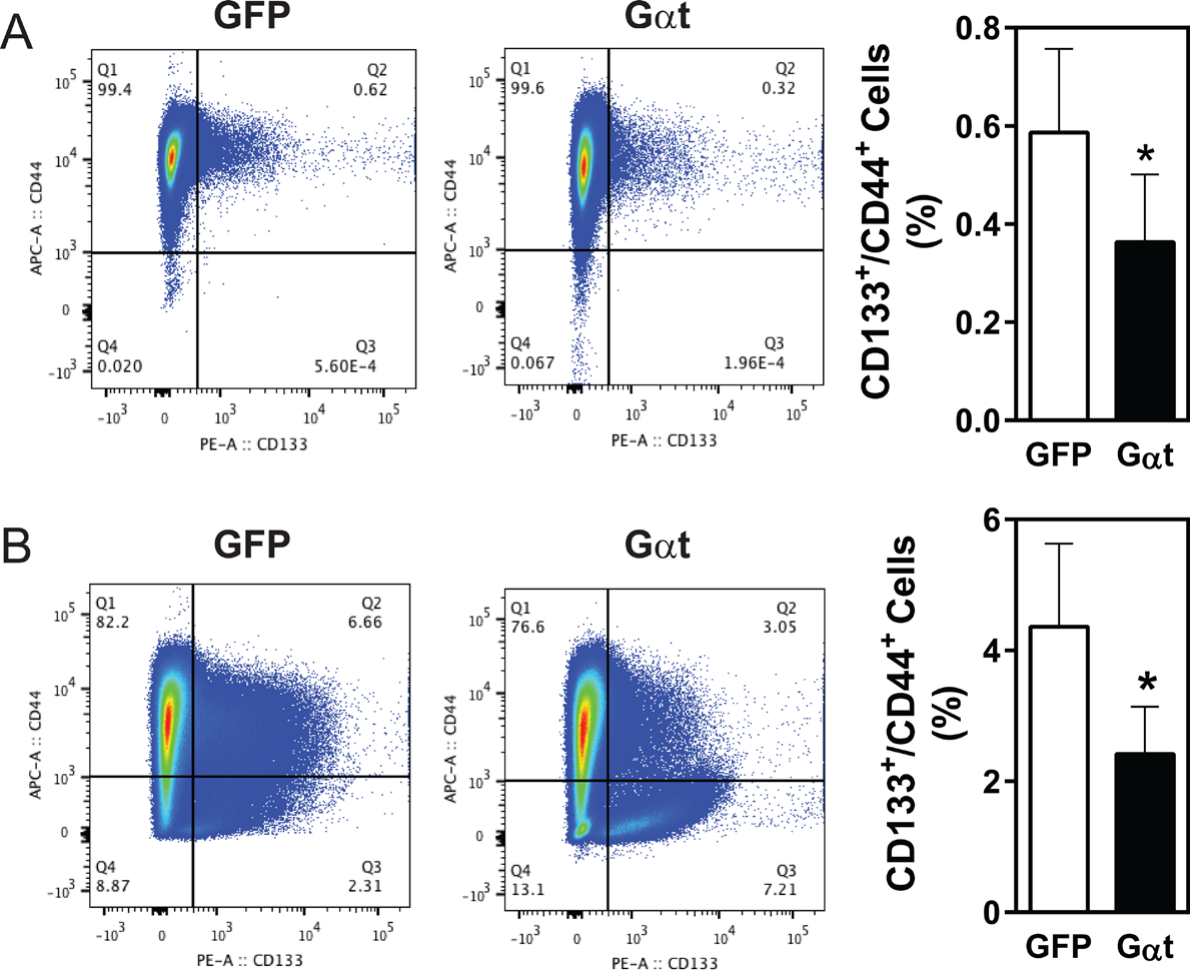
Inhibition of Gβγ signaling decreases the CD133^+^/CD44^+^ populations in PC3 cells and PC3 xenograft tumors Single cells were prepared from PC3 cells grown in 2D culture (A) or PC3 xenograft tumors derived from mice inoculated with 20, 000 cells/mouse, as indicated in Table 3 (B). Flow cytometric analysis was then performed, to track CD133^+^/CD44^+^ cells in live PC3 cells (A) or CD31^−^/CD45^−^/Ter119^−^ cells (B). Representative flow cytometric data and quantitative data are shown. *p < 0.05 *versus* GFP, *n* = 3–4.

The cell surface markers, CD133 and CD44, are commonly used to isolate prostate cancer cells enriched for CSCs [43–45]. Flow cytometry analysis of PC3 cells, grown under monolayer culture conditions, identified fewer than 1% as CD133^+^/CD44^+^ (Figure 8A), and tumorsphere-forming conditions did not stimulate their outgrowth (data not shown). Nevertheless, in established PC3 tumors in nude mice, the CD133^+^/CD44^+^ population was significantly higher than in the cultured cells (4.2 % vs 0.6 %; Figure 8B). This increase in CD133^+^/CD44^+^ the population is unlikely due to contamination of stromal cells because CD31^+^/CD45^+^/Ter119^+^ cells were excluded from analysis. In both cultured PC3 cells and established tumors, blocking Gβγ signaling reduced the CD133^+^/CD44^+^ population by 50% (Figure 8A–8B).

### Blocking Gβγ signaling enhances the therapeutic efficacy of paclitaxel

**Figure 9:**
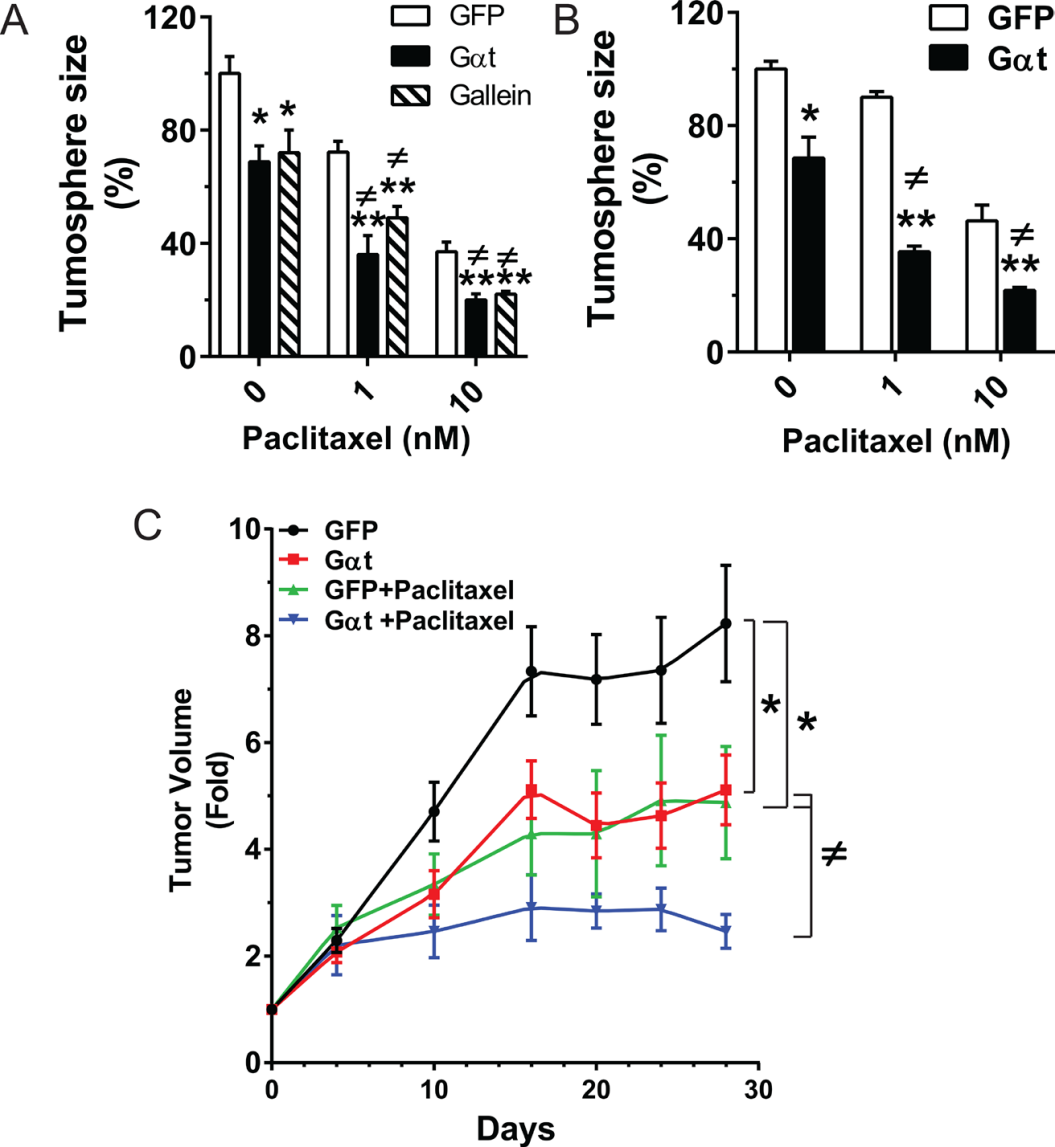
Blocking Gβγ signaling increases the therapeutic efficacy of paclitaxel *in vitro* and *in vivo* (**A–B**) single cells prepared from the third passages of PC3 and DU145 cells grown in ultralow-adhesive plates were plated in Matrigel in 48-well plates (1,000 cells/well), and treated with the indicated concentration of paclitaxel, with and without gallein (20 μM) or doxycycline (1 μg/ml) for 8 days. The tumorsphere size was quantified as in Figure 2. Data are expressed as percentage of the size of tumorspheres from GFP-expressing cells in the absence of paclitaxel treatment. *, **p < 0.05 and 0.01 vs GFP; ^≠^*p* < 0.05 vs Gαt in the absence of paclitaxel. *N* = 3. (C) Mice (*n* = 6–7) were subcutaneously implanted with PC3 cells expressing inducible GFP or Gαt. Tumor growth was measured by caliper. When tumor size reached ~300 mm^3^, mice were fed doxycycline-containing diets and treated with vehicle or paclitaxel (10 mg/kg, i.p., twice per week). *, **p < 0.05 and 0.01 vs GFP; ^≠^*p* < 0.05 vs Gαt or GFP+paclitaxel.

CSCs contribute to prostate cancer resistance to chemotherapy [10, 43]. Since targeting Gβγ signaling inhibits the self-renewal activity and tumorigenicity of prostate cancer CSCs, we tested whether blocking Gβγ signaling sensitizes prostate cancer cells to the chemotherapeutic agent, paclitaxel. We first evaluated the effect of mono- or combinational therapy on the tumorsphere-forming capacity of PC3 and DU145 cells *in vitro*. (To quantify tumorsphere formation, tumorspheres were cultured on Matrigel rather than ultralow-adhesive plates, because dead cells aggregate under the latter condition, obscuring tumorsphere size and number.) Paclitaxel alone inhibited PC3 and DU145 tumorsphere formation in a dose-dependent manner (Figure 9A–9B); and the inhibitory effect was significantly enhanced by simultaneously blocking Gβγ signaling (Figure 9A–9B). The effect of combining Gβγ blockade with paclitaxel was greater than either treatment alone (Figure 9A–9B), suggesting synergistic inhibition.

To translate our findings to an *in vivo* model, PC3 xenograft tumors were established from GFP- or Gαt-expressing cells isolated from tumorspheres grown on ultralow-adhesive plates, in the absence of doxycycline. When the tumors reached a size of ~300 mm^3^, the mice were fed doxycycline-containing diets, to induce GFP or Gαt expression. Silmutaneously, mice were given a sub-maximal dose of vehicle control or paclitaxel. As expected, paclitaxel treatment or Gαt expression alone inhibited tumor growth by ~1.6 fold. When combined with Gαt expression, paclitaxel decreased the rate of tumor growth by ~4-fold (Figure 9C), suggesting a possible synergistic effect.

## DISCUSSION

A growing body of evidence shows GPCRs play a critical role in prostate cancer cell proliferation and dissemination [46–48]. Our studies demonstrate that GPCR signals that drive prostate cancer growth and metastasis converge at one signaling hub—the Gβγ subunits. These findings are consistent with previous reports that in PC3 cells, inhibiting Gβγ signaling by overexpressing the Gβγ scavenger, the C-terminal tail of G protein-coupled receptor kinase 2, alleviated proliferation *in vitro* and subcutaneous tumor formation, in nude mice [35]. Moreover, we provide the first evidence that Gβγ signaling is a central regulator of the self-renewal activity and tumorigenicity of prostate CSCs. Importantly, in our studies, targeting Gβγ signaling inhibited the tumorigenicity of prostate CSCs and sensitized tumors to chemotherapy.

Our studies showed that blocking Gβγ signaling does not affect the growth of the non-transformed prostate epithelial cell line, RPWE1. Unlike in cancer cell lines, in RWPE1 cells, GPCRs stimulate AKT activation through a Gβγ-independent pathway. This suggests that Gβγ signaling may be dispensable for normal cell growth. Thus, GPCRs may drive normal and cancer cell growth through different mechanisms, and targeting Gβγ signaling may be an effective strategy to selectively target tumor cells, while sparing normal cell function. Interestingly, the effect of Gβγ inhibition on cell growth can be mimicked by a specific Gβγ inhibitor, gallein, or pertussis toxin treatment, indicating that the Gβγ is predominantly liberated from activated Gi/o proteins that couple to many GPCRs overexpressed in prostate cancer cells, such as CXCR4 and LPA receptors [13, 32, 36].

Tumor metastasis is the major cause of patient mortality [2], and bone is the primary site of prostate cancer metastasis [49]. Clinically, the bones that are most commonly involved are vertebrae, sternum, pelvic bones, ribs, and femurs [50]. In the intracardiac injection mouse model of prostate cancer metastasis, mandible, tibia and femur are most commonly affected [51]. Interestingly, inhibiting Gβγ signaling largely blocks tumor formation in tibial and femurs but has little effet in the mandible, suggesting GPCR or Gβγ signaling affects bone metastasis differently at different metastasis sites.

Increasing evidence indicates that prostate cancer progresses from a local to a metastatic disease, in part because of a reservoir of CSCs [9–11]. These cells display the stem-cell-like ability to self-renew and then generate diverse cells that constitute a tumor. Gβγ signaling appears to be critical for maintaining prostate CSCs. Inhibiting Gβγ signaling reduces the number and size of tumorspheres grown from PC3, DU145 and 22Rv1 on serial passages *in vitro*. And in a limiting dilution, tumor-formation assay, PC3 cells generated far fewer tumors under Gβγ blockade through Gαt expression in tumor cells or systemic administration of the specific inhibitor of Gβγ, gallein. One caveat to these results is that it is difficult to separate general effects that Gβγ blockade may have on prostate cancer cell growth from more specific effects on CSCs. However, Gβγ blockage decreases the frequency of CD133^+^/CD44^+^ populations in PC3 cells *in vitro* and in tumor xenografts, supporting the idea that Gβγ blockade does affect CSCs.

Taxanes, such as docetaxel and cabazitaxel, are first-line chemotherapeutic agents for metastatic CRPC patients [52, 53]. After initial responses, patients almost always acquire resistance to taxane drugs, likely in part because of CSCs [10, 43]. As compared to bulk tumor cells, CSCs are resistant to most chemotherapeutic agents so treatment with taxanes likely enriches for CSCs. This has been considered as a potential source of tumor relapse and resistance. Thus, targeting CSCs should inclued enhancing sensitivity to taxanes. Indeed, our data support this hypothesis. Inhibiting Gβγ signaling sensitizes PC3-derived tumorspheres to paclitaxel, both *in vitro* and *in vivo*, although further experiments would be necessary to determine if combining these agents have additive versus synergistic effects. These findings imply that aberrant GPCR signaling, through Gβγ, in CSCs may contribute to resistance to paclitaxel. How Gβγ activates the downstream signaling pathways to enhance CSC paclitaxel sensitivity remains to be determined. However, signaling molecules activated by Gβγ (e.g., PI3K/AKT, MAPK, and STAT3) have been shown to regulate sensitivity to chemotherapeutic reagents, including taxanes [9, 31, 32, 41, 52, 54, 55]. Thus, Gβγ likely increases the sensitivity of prostate CSCs to taxanes and tumor progression through diverse signaling pathways.

In summary, we presented clear evidence that Gβγ is a convergence point for multiple GPCRs that promote prostate cancer growth and metastasis. Moreover, we showed that Gβγ signaling is critical for maintaining the tumorigenicity of reseident CSCs. Thus, targeting Gβγ may be a useful approach for eliminating prostate cancer CSCs, to halt tumor progression and augment sensitivity to chemotherapy.

## MATERIALS AND METHODS

### Reagents

LPA, EGF, IGF and PTx were from Sigma. Human SDF1-α was from Pepro Tech. Growth-factor-reduced Matrigel was from BD Biosciences. Rabbit anti-AKT, mouse anti-phospho-AKT, rabbit anti-ERK1/2, and mouse anti-phospho-ERK1/2 antibodies were from Cell Signaling Technology. Mouse anti-mortalin was from NeuroMab. Mouse anti-Gαt was a gift from Dr. Heidi Hamm (Vanderbilt University). Human allophycocyanin-conjugated CD44 was from BD Biosciences, and human phycoerythrin-conjugated CD133 was from Miltenyl Biotech. Paclitaxel was from LC Laborateries. Gallein was from TCI America.

### Lentiviral production

The pSLIK lentiviral vectors for tetracycline inducible EGFP and Gαt expression were described previously [37, 38]. Lentiviruses were generated by transfecting 293FT cells with the pSLIK vectors together with packaging vectors pMDL and pSPAX2 (Addgene).

### Cell culture and establishment of stable cell lines

The human prostate cancer cell lines, PC3, DU145, and 22RV1, and the normal prostate epithelial cell line RWPE1, were obtained from ATCC. PC3 and DU145 cells were cultured in DMEM (Invitrogen) supplemented with 10% fetal bovine serum (FBS), while 22Rv1 cells were cultured in RPMI-1640 (Life Technologies) supplemented with 10% FBS. RWPE1 cells were cultured in keratinocyte serum-free medium (Life Technologies) supplemented with 0.05 mg/ml bovine pituitary extract and 5 ng/ml EGF. PC3, DU145 and 22Rv1 cells were transduced with lentivirus prepared from the pLenti PGK Neo vector to express firefly luciferase and selected with G418 (0.5 mg/ml).

The PC3, DU145, 22Rv1 and RWPE1 cells were transduced with pSLIK lentiviruses encoding Tet-inducible EGFP or Gαt and selected with hygromycin (200–500 μg/ml) to establish stable cell lines.

### Western blotting analysis

Cells or tissues were lysed in RIPA buffer (50 mM Tris-HCl, pH7.4, 150 mM NaCl, 1% Nonidet P-40, 0.1% SDS and 1 mM EDTA) containing protease and phosphatase inhibitors. Western blotting was performed as reported, using an Odyssey infrared imaging system (Li-Cor Biosciences) or chemiluminescent substrates for visualization [37, 38].

### Cell growth in two- and three-dimensional cultures

Cell growth was assessed in 2-dimensitonal (2D) monolayer culture by using XTT (2, 3-Bis (2-methoxy-4-nitro-5-sulphophenyl)-2H-tetrazolium-5-carboxanilide). Cells stably expressing EGFP or Gαt were seeded in 96-well plates (2000 cells/well) in the growth medium containing 10% FBS for 24 hr, and then treated with doxycycline (1 μg/ml) to induce GFP or Gαt expression. Cell growth was assayed either daily or every two days by the addition of XTT compound followed by absorbance measurement at 450 nM in a microplate reader [37, 38].

To assess the ability of prostate cancer cells to form colonies in three-dimensional cultures, cells were suspended in complete growth medium, supplemented with 2% growth factor-reduced Matrigel (BD Biosciences) and cultured on top of a thin layer of Matrigel in 8-well chamber slides (1,000 cells/well) [37, 38]. Doxycycline was added to cells the next day; and the medium was replaced every 3 days. On day eight of culture, cells were assessed via microscopy and multiple images were taken. Colony sizes were analyzed by ImageJ software.

### BrdU incorporation assay

Three days after treatment with doxycycline to induce GFP or Gαt expression, prostate cancer cells, grown on coverslips in a 24-well plate, were serum starved overnight. A BrdU incorporation assay was then performed for 16 hours, as described [38].

### Cell migration assay

PC3, DU145, and 22RV1 cells were induced to express GFP or Gαt for three days. After serum starvation overnight, cells were detached from plates using 2 mM EDTA/PBS. After washing with serum-free DMEM twice, cells were resuspended in serum-free DMEM and subjected to transwell migration assay for 4 h at 37°C, as described previously [37, 38].

### Xenograft mouse models

All animal studies were conducted in accordance with an Institutional Animal Care and Use Committee- approved protocol at the University of Iowa. 6 to 8-week-old, male, nude mice were used for studies. PC3 or 22Rv1 cells expressing inducible GFP or Gαt were implanted into mice either by injection into the left ventricle (2 × 10^5^ cells in 100 μl PBS) or the dorsolateral prostate lobe (2 × 10^5^ cells in 20 μl PBS). 21 days post-injection, mice were fed a doxycycline-containing diet (TD.01306, Harlan laboratories, 625 mg/kg) continuously, to induce GFP and Gαt expression. Tumor progression was monitored weekly by BLI. Mice were euthanized when they lost more than 15% of their body weight. The formation of tumors in various tissues was determined by post-mortem *ex-vivo* BLI. The formation of bone tumors was confirmed by X-ray and histological analysis by H&E staining.

### BLI

Mice were anaesthetized and injected retroorbitally with 100 μl D-luciferin (15 μg/ml in PBS). BLI was performed using a Xenogen IVIS200 system. The bioluminescent data were analyzed by the software Living Image (Xenogen) and expressed as photon flux (photons/s/cm^2^/sr) [37, 38].

### Tumorsphere culture

Single PC3, DU145, and 22RV1 cells, expressing inducible GFP or Gαt, were plated (1,000 cells/ml) on ultralow-attachment, 6-well or 10-cm plates, coated with poly(2-hydroxyethl methacrylate (5 mg/ml in 95% ethanol) in serum-free prostate epithelial cell basal medium (Lonza) supplemented with 4 μg/ml insulin, 20 ng/ml EGF, 20 ng/ml FGF, and B27 (Life Technology) as described previously [41]. After 7 to 14 days of culture, the number of tumorspheres was quantified under an inverted microscope. To determine the size of tumorspheres, images were taken at 5–10 randomly chosen areas and analyzed by ImageJ software. Spheres were dissociated with Accutase (Invitrogen) to generate single cell suspension for subsequent expansion and flow cytometry analysis.

To quantify the sensitivity of GFP- or Gαt-expressing tumorspheres to paclitaxel, third passages were dispersed to single cells and cultured in Matrigel, as described above. Cells were then treated with different concentrations of paclitaxel together with doxycycline for 8 days.

### Tumorigenicity of prostate cancer stem cells *in vivo*

PC3 cells expressing inducible GFP or Gαt were maintained in tumorsphere culture, in the absence of doxycycline for three generations. Single cells were prepared from the third generation of tumorspheres and various numbers (50,000, 20,000, and 10,000 cells) were subcutaneously injected into the upper and lower flanks of nude mice. Following injection, mice were immediately fed doxycycline-containing diets to induce GFP and Gαt expression. To evaluate the effect of Gβγ inhibitor, gallein, on tumor formation, mice implanted with GFP expressing PC3 cells were treated with gallein (10 mg/kg in PBS, i.p., twice daily). Tumor formation was monitored for 8 weeks by palpation and caliper measurement.

To determine the sensitivity of tumorsphere-derived xenograft tumors to the combination of paclitaxel treatment and the blockage of Gβγ signaling, 1 × 10^6^ of PC3 cells dissociated from the third passages of GFP- or Gαt- expressing tumorsheres formed in the absence of doxycycline treatment were injected subcutaneously to 6 to 8-week-old male mice. Primary tumor growth was initially monitored by bioluminescence imaging. Once tumors became palpable, their growth was recorded with caliper measurement of tumor length (L) and width (W) every 5 days. Tumor volume was calculated by the formula of length × width^2^ × 0.5. When the tumors reached a size of ~300 mm^3^, mice were fed doxycyline-containing diet and treated with paclitaxel (10 mg/kg, i.p., twice per week) or vehicle.

### Flow cytometry analysis

Cells were dissociated from two-dimensional cultures using Accutase. To dissociate cells from xenograft tumors, tumors were cut into small pieces and digested with collagenase 3 (Collagenase Type3, Worthington) at 37^°^C for 2–3 hours as reported [41]. Cells were resuspended in PBS containing 2% FBS and 1mM EDTA and stained with APC-conjugated anti-CD44 and PE-conjugated anti-CD133 antibodies at 4°C for 30 min. To exclude stromal cells in tumor samples, dissociated cells were also stained first with biotin-labeled anti mouse CD31, CD45 and Ter119, followed by PE/Cy7-conjugated streptavidin. Samples were analyzed on a LSR II violet flow cytometer (Becton Dickinson). At least 1 × 10^6^ viable cell events were collected per sample with appropriate negative and single-color positive staining controls. For tumor samples, CD31^−^/CD45^−^/Ter119^−^ cells were analyzed for CD44 and CD133 expression.

### Immunohistochemistry

Resected xenograft tumors were fixed with 4% paraformaldehyde, dehydrated with 20% sucrose solution, and embedded in paraffin. Tissues were sectioned at 10-mm intervals, deparaffinized and stained with rabbit anti-Ki67 (1:100, GenTex) or anti-cleaved caspase 3 (1:100, Cell Signaling Technology) antibodies, followed by the HRP-conjugated goat anti rabbit IgG secondary antibody (1:200) using the ultra-sensitive ABC peroxidase staining kit (Pierce) [37]. At least 5 images per section at random fields were taken by a Leica ICC50HD microscope using 10x lens and analyzed by the Image J software.

### Statistical analysis

Survival curves were analyzed according to the Kaplan-Meier method using the Graphpad Prism 6 Software, and the differences between curves were evaluated by the log-rank test. To compare xenograft tumor growth rates after doxycycline and/or paclitaxel treatment, the tumor size was plotted against time. A linear regression analysis was performed using the Graphpad Prism 6 Software, to obtain the slopes, and the difference in the slope values were evaluated by the analysis of covariance. Data are expressed as mean ± S.E.M. Means between two groups were compared with a two-tailed, unpaired Student's *t* test, while comparisons of means from multiple groups with each other or against one control group were analyzed with one-way ANOVA, followed by Tukey's range test. A *p* value of less than 0.05 was considered to be statistically significant.

## References

[1] Haas GP, Delongchamps N, Brawley OW, Wang CY, de la Roza G (2008). The worldwide epidemiology of prostate cancer: perspectives from autopsy studies. Can J Urol.

[2] Cheville JC, Tindall D, Boelter C, Jenkins R, Lohse CM, Pankratz VS, Sebo TJ, Davis B, Blute ML (2002). Metastatic prostate carcinoma to bone: clinical and pathologic features associated with cancer-specific survival. Cancer.

[3] Watson PA, Arora VK, Sawyers CL (2015). Emerging mechanisms of resistance to androgen receptor inhibitors in prostate cancer. Nat Rev Cancer.

[4] Zong Y, Goldstein AS (2013). Adaptation or selection—mechanisms of castration-resistant prostate cancer. Nat Rev Urol.

[5] Saylor PJ (2013). Prostate cancer: The androgen receptor remains front and centre. Nat Rev Clin Oncol.

[6] Yuan X, Balk SP (2009). Mechanisms mediating androgen receptor reactivation after castration. Urol Oncol.

[7] Lorenzo GD, Bianco R, Tortora G, Ciardiello F (2003). Involvement of growth factor receptors of the epidermal growth factor receptor family in prostate cancer development and progression to androgen independence. Clin Prostate Cancer.

[8] Raj GV, Barki-Harrington L, Kue PF, Daaka Y (2002). Guanosine phosphate binding protein coupled receptors in prostate cancer: a review. J Urol.

[9] Rybak AP, Bristow RG, Kapoor A (2015). Prostate cancer stem cells: deciphering the origins and pathways involved in prostate tumorigenesis and aggression. Oncotarget.

[10] Jaworska D, Krol W, Szliszka E (2015). Prostate Cancer Stem Cells: Research Advances. Int J Mol Sci.

[11] Ojo D, Lin X, Wong N, Gu Y, Tang D (2015). Prostate Cancer Stem-like Cells Contribute to the Development of Castration-Resistant Prostate Cancer. Cancers (Basel).

[12] Dubrovska A, Elliott J, Salamone RJ, Telegeev GD, Stakhovsky AE, Schepotin IB, Yan F, Wang Y, Bouchez LC, Kularatne SA, Watson J, Trussell C, Reddy VA (2012). CXCR4 expression in prostate cancer progenitor cells. PLoS One.

[13] Daaka Y (2004). G proteins in cancer: the prostate cancer paradigm. Sci STKE.

[14] Oldham WM, Hamm HE (2008). Heterotrimeric G protein activation by G-protein-coupled receptors. Nat Rev Mol Cell Biol.

[15] Dorsam RT, Gutkind JS (2007). G-protein-coupled receptors and cancer. Nat Rev Cancer.

[16] Lappano R, Maggiolini M (2011). G protein-coupled receptors: novel targets for drug discovery in cancer. Nat Rev Drug Discov.

[17] Wang J, Lu Y, Koch AE, Zhang J, Taichman RS (2008). CXCR6 induces prostate cancer progression by the AKT/mammalian target of rapamycin signaling pathway. Cancer Res.

[18] Wang J, Shiozawa Y, Wang Y, Jung Y, Pienta KJ, Mehra R, Loberg R, Taichman RS (2008). The role of CXCR7/RDC1 as a chemokine receptor for CXCL12/SDF-1 in prostate cancer. J Biol Chem.

[19] Wang J, Sun Y, Song W, Nor JE, Wang CY, Taichman RS (2005). Diverse signaling pathways through the SDF-1/CXCR4 chemokine axis in prostate cancer cell lines leads to altered patterns of cytokine secretion and angiogenesis. Cell Signal.

[20] Hirbe AC, Morgan EA, Weilbaecher KN (2010). The CXCR4/SDF-1 chemokine axis: a potential therapeutic target for bone metastases?. Curr Pharm Des.

[21] Barki-Harrington L, Bookout AL, Wang G, Lamb ME, Leeb-Lundberg LM, Daaka Y (2003). Requirement for direct cross-talk between B1 and B2 kinin receptors for the proliferation of androgen-insensitive prostate cancer PC3 cells. Biochem J.

[22] Guo R, Kasbohm EA, Arora P, Sample CJ, Baban B, Sud N, Sivashanmugam P, Moniri NH, Daaka Y (2006). Expression and function of lysophosphatidic acid LPA1 receptor in prostate cancer cells. Endocrinology.

[23] Gohji K, Kitazawa S, Tamada H, Katsuoka Y, Nakajima M (2001). Expression of endothelin receptor a associated with prostate cancer progression. J Urol.

[24] Nelson J, Bagnato A, Battistini B, Nisen P (2003). The endothelin axis: emerging role in cancer. Nat Rev Cancer.

[25] Xie Y, Gibbs TC, Mukhin YV, Meier KE (2002). Role for 18: 1 lysophosphatidic acid as an autocrine mediator in prostate cancer cells. J Biol Chem.

[26] Vindrieux D, Escobar P, Lazennec G (2009). Emerging roles of chemokines in prostate cancer. Endocr Relat Cancer.

[27] Singh RK, Lokeshwar BL (2009). Depletion of intrinsic expression of Interleukin-8 in prostate cancer cells causes cell cycle arrest, spontaneous apoptosis and increases the efficacy of chemotherapeutic drugs. Mol Cancer.

[28] Zhang S, Qi L, Li M, Zhang D, Xu S, Wang N, Sun B (2008). Chemokine CXCL12 and its receptor CXCR4 expression are associated with perineural invasion of prostate cancer. J Exp Clin Cancer Res.

[29] Carducci MA, Saad F, Abrahamsson PA, Dearnaley DP, Schulman CC, North SA, Sleep DJ, Isaacson JD, Nelson JB (2007). A phase 3 randomized controlled trial of the efficacy and safety of atrasentan in men with metastatic hormone-refractory prostate cancer. Cancer.

[30] Nelson JB, Love W, Chin JL, Saad F, Schulman CC, Sleep DJ, Qian J, Steinberg J, Carducci M (2008). Phase 3, randomized, controlled trial of atrasentan in patients with nonmetastatic, hormone-refractory prostate cancer. Cancer.

[31] Smrcka AV, Lehmann DM, Dessal AL (2008). G protein betagamma subunits as targets for small molecule therapeutic development. Comb Chem High Throughput Screen.

[32] Smrcka AV (2008). G protein betagamma subunits: central mediators of G protein-coupled receptor signaling. Cell Mol Life Sci.

[33] Daaka Y (2002). Mitogenic action of LPA in prostate. Biochim Biophys Acta.

[34] Kue PF, Taub JS, Harrington LB, Polakiewicz RD, Ullrich A, Daaka Y (2002). Lysophosphatidic acid-regulated mitogenic ERK signaling in androgen-insensitive prostate cancer PC-3 cells. Int J Cancer.

[35] Bookout AL, Finney AE, Guo R, Peppel K, Koch WJ, Daaka Y (2003). Targeting Gbetagamma signaling to inhibit prostate tumor formation and growth. J Biol Chem.

[36] Kue PF, Daaka Y (2000). Essential role for G proteins in prostate cancer cell growth and signaling. J Urol.

[37] Tang X, Sun Z, Runne C, Madsen J, Domann F, Henry M, Lin F, Chen S (2011). A critical role of Gbetagamma in tumorigenesis and metastasis of breast cancer. J Biol Chem.

[38] Ye Y, Tang X, Sun Z, Chen S (2016). Upregulated WDR26 serves as a scaffold to coordinate PI3K/ AKT pathway-driven breast cancer cell growth, migration, and invasion. Oncotarget.

[39] Faure M, Voyno-Yasenetskaya TA, Bourne HR (1994). cAMP and beta gamma subunits of heterotrimeric G proteins stimulate the mitogen-activated protein kinase pathway in COS-7 cells. J Biol Chem.

[40] Katada T (2012). The inhibitory G protein G(i) identified as pertussis toxin-catalyzed ADP-ribosylation. Biol Pharm Bull.

[41] Dubrovska A, Kim S, Salamone RJ, Walker JR, Maira SM, Garcia-Echeverria C, Schultz PG, Reddy VA (2009). The role of PTEN/Akt/PI3K signaling in the maintenance and viability of prostate cancer stem-like cell populations. Proc Natl Acad Sci USA.

[42] Lang SH, Frame FM, Collins AT (2009). Prostate cancer stem cells. J Pathol.

[43] Dubrovska A, Elliott J, Salamone RJ, Kim S, Aimone LJ, Walker JR, Watson J, Sauveur-Michel M, Garcia-Echeverria C, Cho CY, Reddy VA, Schultz PG (2010). Combination therapy targeting both tumor-initiating and differentiated cell populations in prostate carcinoma. Clin Cancer Res.

[44] Richardson GD, Robson CN, Lang SH, Neal DE, Maitland NJ, Collins AT (2004). CD133, a novel marker for human prostatic epithelial stem cells. J Cell Sci.

[45] Collins AT, Berry PA, Hyde C, Stower MJ, Maitland NJ (2005). Prospective identification of tumorigenic prostate cancer stem cells. Cancer Res.

[46] Gibbs TC, Rubio MV, Zhang Z, Xie Y, Kipp KR, Meier KE (2009). Signal transduction responses to lysophosphatidic acid and sphingosine 1-phosphate in human prostate cancer cells. Prostate.

[47] Rodriguez M, Siwko S, Zeng L, Li J, Yi Z, Liu M (2016). Prostate-specific G-protein-coupled receptor collaborates with loss of PTEN to promote prostate cancer progression. Oncogene.

[48] Dbouk HA, Vadas O, Shymanets A, Burke JE, Salamon RS, Khalil BD, Barrett MO, Waldo GL, Surve C, Hsueh C, Perisic O, Harteneck C, Shepherd PR (2012). G Protein-Coupled Receptor-Mediated Activation of p110beta by Gbetagamma Is Required for Cellular Transformation and Invasiveness. Sci Signal.

[49] Bubendorf L, Schopfer A, Wagner U, Sauter G, Moch H, Willi N, Gasser TC, Mihatsch MJ (2000). Metastatic patterns of prostate cancer: an autopsy study of 1,589 patients. Hum Pathol.

[50] Roudier MP, True LD, Higano CS, Vesselle H, Ellis W, Lange P, Vessella RL (2003). Phenotypic heterogeneity of end-stage prostate carcinoma metastatic to bone. Hum Pathol.

[51] Drake JM, Gabriel CL, Henry MD (2005). Assessing tumor growth and distribution in a model of prostate cancer metastasis using bioluminescence imaging. Clin Exp Metastasis.

[52] Kroon J, Kooijman S, Cho NJ, Storm G, van der Pluijm G (2016). Improving Taxane-Based Chemotherapy in Castration-Resistant Prostate Cancer. Trends Pharmacol Sci.

[53] Cooper BT, Sanfilippo NJ (2015). Concurrent chemoradiation for high-risk prostate cancer. World J Clin Oncol.

[54] Liu Z, Zhu G, Getzenberg RH, Veltri RW (2015). The Upregulation of PI3K/Akt and MAP Kinase Pathways is Associated with Resistance of Microtubule-Targeting Drugs in Prostate Cancer. J Cell Biochem.

[55] Yuen JW, Poon LS, Chan AS, Yu FW, Lo RK, Wong YH (2010). Activation of STAT3 by specific Galpha subunits and multiple Gbetagamma dimers. Int J Biochem Cell Biol.

